# Auditory comprehension performance of college students with and without sport concussion on Computerized-Revised Token Test Subtest VIII

**DOI:** 10.2217/cnc-2016-0024

**Published:** 2017-05-08

**Authors:** Anthony P Salvatore, Michael Cannito, Heather E Brassil, Edina R Bene, Bess Sirmon-Taylor

**Affiliations:** 1 Department of Rehabilitation Sciences, University of Texas at El Paso, El Paso, TX 79902, USA; 2 Department of Communicative Disorders, University of Louisiana-Lafayette, Lafayette, LA 70504, USA; 3 School of Communication Sciences & Disorders, University of Memphis, Memphis, TN 38152, USA

**Keywords:** auditory comprehension, clinical assessment, Computerized-Revised Token Test, concussion, ImPACT test

## Abstract

**Aim:**

Auditory comprehension (AC) and visually assessed cognitive functions were compared in early stage postconcussed (PC) athletes and healthy controls using the Subtest VIII of the Computerized-Revised Token Test (C-RTT) and Immediate Postconcussion Assessment and Cognitive Test (ImPACT).

**Results:**

As compared with healthy controls (n = 30), PC subjects (n = 30) had significantly lower C-RTT efficiency scores (p = 0.018), and lower ImPACT scores; total symptom score (p = 0.000.), verbal memory (p = 0.000), visual memory (p = 0.000), visual motor speed (p = 0.000) and reaction time (p = 0.004) in this post-test only matched subject design. Impulse Control was not significant (p = 0.613). Multiple regression and ANOVA indicated an association with reaction time only (p = 0.012) for the PC subjects. After controlling for reaction time, a significant difference in AC remained.

**Conclusion:**

The relationship between AC and other visually assessed cognitive functions was inconsistent suggesting that the C-RTT and ImPACT assessed different functional systems.

Sports-related concussion has received increased attention over the past decade. Although this is most evident in professional football, concussion is also a serious health issue in high school and collegiate student-athletes. According to the Centers for Disease Control, there are an estimated 300,000 sports-related concussions that occur annually [1]. A concussion is defined as a blow to the body or head that causes a jarring motion of the brain which, in turn, results in a dysfunction of the brain [2]. Concussions can be characterized by difficulties with memory, concentration, headache, dizziness, fatigue, light and noise sensitivity, irritability, sadness, anxiety and sleep-related symptoms. A concussion, a subtype of mild traumatic brain injury (mTBI), is characterized by a shift in ionic status, disruption in energy metabolism, diminished cerebral blood flow and impaired neural transmission [3]. MTBI may result in diffuse disruption of neural function of the brain. This neural dysfunction results in signs and symptoms which may not be evident immediately postinjury but may persist for days and months postconcussion (PC). Focal and diffuse disruption to the brain characterizes a traumatic brain injury [4]. The prefrontal or frontolimbic structures are considered most vulnerable in a traumatic brain injury. Disruption or damage to these areas is associated with cognitive-communication impairments such as auditory comprehension [5,6]. Holland noted that auditory comprehension deficits were common characteristics of aphasia and closed head injuries [7].

The clinical assessment of a student-athlete following a concussion should include sufficient information to make decisions about when the individual can return to the classroom as well as practice/play. In addition to an assessment of memory and new learning, the status of auditory comprehension of spoken messages should be examined, given its primary role in the classroom setting. Auditory comprehension is the ability to understand and act on spoken sentences, and is one of several cognitive-communication behaviors potentially impacted by a concussion. Participation in academics, employment, social interactions, activities of daily living and sports is very much dependent upon the ability to understand spoken messages. For example, a college athlete’s classroom participation such as responding to questions, taking notes and understanding lecture material maybe adversely impacted following a sport-related concussion.

An extensive search of the literature showed a paucity of data regarding the status of the cognitive-communication process of auditory comprehension following a sport-related concussion. Murdoch and Theodoros reported deficits in auditory comprehension in a large group of individuals with mild-moderate and severe traumatic brain injuries [8]. Unfortunately, their sample lacked control for etiology and severity. More recently, Whelan, Murdoch and Bellamy reported on the communication impairments associated with mild TBI following an automobile accident, in a 19-year-old female whose performance was evaluated relative to a group of 10 young non-neurologically impaired adults [5]. Compared with the healthy controls (HC), this individual showed multiple cognitive-communicative deficits, for example, in attention, lexical access and manipulation of complex lexical-semantic relationships. These authors concluded that their findings ‘provide some support for the hypothesis of cognitive-communication difficulties following mTBI’, (p.196) [5]. Therefore, investigating a pool of young athletes with concussions may shed light on the presence or absence of cognitive-communication deficits such as auditory comprehension in this population.

Meyers and Rohling [9] reviewed a number of instruments and reported that one instrument, the Token Test [10], a test of auditory comprehension, approached statistical significance (p = 0.058) as part of a battery of tests that successfully differentiated between mild brain injuries (defined as loss of consciousness of less than 5 min) and a noninjured normal control group. The Token Test was developed as a sensitive measure of auditory comprehension in individuals with aphasia. This instrument and its various subsequent modifications have been widely used to assess auditory comprehension of spoken sentences. The test requires an individual to point to or manipulate 20 tokens of varying shapes and colors, according to spoken sentence instructions. The test involves a small vocabulary with variable sentence length and grammatical complexity over each of five parts of the test. Norms for children and adults are provided by Gaddes and Crockett [11], Noll and Lass [12], and Spreen and Benton [13]. The sensitivity of the test to mild and moderate impairment makes it suitable for use in younger PC athletes who are unlikely to have yet accumulated substantial damage or marked cognitive change.

Murdoch, Lewis and Knuepffer recommend the use of the Revised Token Test (RTT) for assessment of comprehension of complex auditory information as part of their linguistic assessment battery for adults with TBI [14]. The RTT and the subsequent Computerized-Revised Token Test (C-RTT) [15]. The subtests assess the accuracy and speed of an individual’s responses to structurally and informationally complex spoken sentences. The most difficult subtest is Subtest VIII which assesses comprehension of complex sentences such as ‘Put the small red circle to the left of the large green square’ [16]. An investigation of the reliability and validity of the C-RTT showed that this test distinguished between an HC group and a group of people with aphasia. Also, the test showed adequate test–retest reliability, which supports its use with people with aphasia [17].

Hinchliffe, Murdoch and Chenery reported that individuals with severe TBI showed impaired processing of higher order spoken sentences that require lexical-semantic manipulations like those found in Subtest VIII of the C-RTT [18]. Furthermore, the Efficiency Score (ES) generated by the C-RTT incorporates two parameters adversely affected by brain damage: accuracy and reaction time. The presence and nature of such a disruption in processing auditory information may add to the knowledge base related to the cognitive-communicative function of individuals who suffer a concussion. For these reasons, the C-RTT Subtest VIII was adopted for clinical use in assessing the nature and extent of sport-related concussion sequelae on auditory comprehension.

Related to the interest regarding the presence or absence of auditory dysfunction in concussed individuals is the question of how concussed individuals process spoken sentences. One assumption is that understanding a sentence requires the use of cognitive resources such as attention and storage capacity. Processing auditory information is a temporal and sequential process that can be described parsimoniously by a unitary store model that views working memory (WM) as the temporary active phase of long-term memory (LTM) processing [19]. In his review of cognition of language and communication, Davis suggests that sentence comprehension takes place within the processing capacity of WM [20]. The process of understanding sentences like ‘Put the small red circle on top of the large green square’ may involve chunking the first phrase and processing it in WM, maintaining the meaning of the verb-adjective-noun in an unintegrated state until the following preposition-adjective-noun are processed, and then both phrases are integrated into LTM. The question is whether a concussion yields diminished cognitive resources such as attention and storage capacity that are necessary to adequately carry out this auditory processing task.

One of the major assessment instruments used for assessing PC status is the Immediate Postconcussion Assessment and Cognitive Test (ImPACT) [21]. The test is normed across gender, level of play and age. This computer-based assessment is administered via visual stimuli, and is available in 21 different languages. The test takes between 25 and 30 min to complete. It assesses the executive functions of attention, processing speed, WM and task vigilance [22]. However, these measurements are based entirely on visual stimuli, and the test does not assess auditory comprehension of spoken sentences.

The purpose of this study was to investigate whether college-age individuals who have experienced concussion demonstrated impaired auditory sentence comprehension performance on Subtest VIII of the C-RTT when compared with a matched group of individuals with no recent history of concussion. Our null hypothesis is that there will not be a statistically significant difference in the ES between the group of HCs and the PC group. It was also of interest to examine relationships between auditory comprehension as measured by the C-RTT ES and other cognitive variables as measured by the ImPACT Test in the performance of athletes with concussion. Our second null hypothesis is that there will be a positive correlation between the two instruments given they are both assessing cognitive abilities. Because both groups were composed of male and female participants the potential difference in performance between the genders was also analyzed. Therefore, our third null hypothesis is that there will not be a statistically significant difference in performance between the genders on the ImPACT and C-RTT tests. Finally, these findings will contribute to improved clinical assessment and management of individuals who experience a sport-related concussion.

## Materials & methods

### Sample & participant selection

The experimental design of this study was a post-test only two-group design. A convenience sample of thirty PC student-athletes who had completed the ImPACT test and the C-RTT Subtest VIII was selected from the files of the University of Texas at El Paso Concussion Management Clinic (UTEP CMC). These athletes had been diagnosed with a sport-related concussion and referred to the CMC by a team athletic trainer or physician. The HC group included 30 student-athletes matched for age, years of education and gender. Twenty-four HC subjects were selected from the files of the UTEP CMC and six matched students were recruited from the student population in the Department of Rehabilitation Sciences-UTEP. Participants from both groups were collegiate-aged individuals involved in a variety of sports, the largest proportion of which were football players (Table 1). Fifty-two of the subjects were active in sports at the varsity and semiprofessional levels, eight subjects participated at the high school level and were physically active at the time of testing.

**Table 1.** **Type of sports and percentages within the groups based on gender, n = 60.**

**Number of participants per sport (n = 60)**	**Postconcussion group (n = 30)**				**Healthy control group (n = 30)**			
**Type of sport**	**Females**	**(%)**	**Males**	**(%)**	**Females**	**(%)**	**Males**	**(%)**
Baseball	0	0	1	5	0	0	1	5

Basketball	0	0	1	5	1	12.5	2	9

Football	0	0	16	72	0	0	9	40

Ice hockey	0	0	4	18	0	0	5	23

Soccer	1	12.5	0	0	3	37.5	2	9

Softball	5	62.5	0	0	1	12.5	0	0

Track and field	1	12.5	0	0	0	0	0	0

Volleyball	1	12.5	0	0	2	25	0	0

Tennis	0	0	0	0	0	0	3	14

Dance	0	0	0	0	1	12.5	0	0

Total	8	100	22	100	8	100	22	100

Inclusion criterion for the HC group was a composite percentile score in the average to superior functional range category [21]. Exclusion criteria for both groups included history of hearing acuity disorder, ADD/ADHD, learning disability, brain surgery, substance abuse, epilepsy, seizures or recorded history of more than three previous concussions. An additional exclusion criterion for HC participants was experiencing a concussion within 13 months prior to the study.

In the HC group, 25 participants (83.3%) reported no history of concussion, 3 (10%) reported a history of one concussion, 1 (3.3%) reported a history of two concussions and 1 (3.3%) reported a history of three concussions. In the PC group, 16 participants (53.3%) reported no prior history of concussion, 7 (23.3%) reported a history of one concussion, 4 (13.3%) reported a history of two concussions and 3 (10%) reported a history of three concussions prior to their current concussion.

Within the PC group, a total of six participants (20%) reported a brief loss of consciousness after their injury and seven participants (23.3%) reported post-traumatic amnesia. In the PC group, a total of 23 (76.6%) of the participants had been hit on the head: 6 anteriorly; 7 posteriorly; 7 on the left side of head; and 3 on the right side of head. One participant reported a whiplash injury without contact to the head, and there was no information regarding head contact in the records for six of the participants.

### Procedures & settings

Before the test administration, written informed consent was obtained from each of the participants in both groups. The investigation was approved by the Institutional Review Board at the UTEP. Upon referral to the CMC at UTEP, each participant was administered the ImPACT test followed by Subtest VIII of the C-RTT, both via computer. In addition, an in-depth health history was obtained as part of the first PC assessment. The HC group was administered the ImPACT test and the C-RTT Subtest VIII in the same sequence as the PC individuals.

All testing was carried out in a 10 × 12 feet sound-proof room. The keyboard and monitor were located on separate equipment racks, and the participants were seated comfortably in front of the monitor. The C-RTT sentences were presented free-field via loud speakers at a comfortable listening level as determined by each participant. The listening level for the spoken sentence, measured with a hand-held sound level meter, varied between 45 and 50 db, depending upon the preference of the participant. Each participant sat approximately 2 feet from the free standing speakers. Prior to presenting the test, each participant was administered a short training module to ensure that the participant was able to match colors, shapes and manipulate the mouse properly. After this brief training, Subtest VIII of the C-RTT was presented. Following the presentation of each spoken sentence, the participant responded by moving the mouse from a standard position on the monitor screen to the first object, then clicking and dragging the object to the position requested. The standardized instructions for C-RTT Subtest VIII administration are attached as an Appendix.

### Measures

Two tests were completed by each participant administered in the following sequence: ImPACT test (Version 2) and the C-RTT Subtest VIII.

#### ImPACT test version 2

The ImPACT test was designed to measure PC cognitive changes. The entire test uses only visual stimuli, and is comprised of a series of six tests that together provide five composite scores for verbal memory, visual memory, visual motor speed and reaction time and impulse control. Total symptom score was also measured. The ImPACT includes a 22-item concussion symptom inventory scored on a 7-point Likert-type scale from 0 (‘none’) to 6 (‘most severe’). Table 2 shows the composite scores by group.

**Table 2.** **Descriptive statistics of ImPACT composite scores, total symptom score and cognitive efficiency index score for the postconcussion group (n = 30) and healthy control group (n = 30).**

**ImPACT scores**	**Postconcussion**		**Healthy control**	
	**Mean**	**SD**	**Mean**	**SD**
Verbal memory composite	79.80	15.45	92.2	5.4

Visual memory composite	63.60	17.65	79.97	8.51

Reaction time composite	0.64	0.22	0.55	0.04

Visual motor speed	36.67	6.95	43.42	5.31

Impulse control composite	5.17	3.35	4.47	2.33

Total symptom score	18.20	18.2	1.83	2.61

#### C-RTT Subtest VIII

Following the administration of the ImPACT test, Subtest VIII of the C-RTT was administered. Subtest VIII tests comprehension of relatively demanding spoken sentences including prepositional/locational phrases such as ‘in front of’, ‘behind’, ‘above’, ‘below’, ‘to the left’ or ‘to the right’. The digitally recorded sentences are presented by the C-RTT software, allowing for standardized test administration. Participant responses were made via the mouse and recorded and scored automatically as part of the C-RTT software using a multidimensional scoring system ranging from 1 to 15 (see Table 3). The multidimensional scoring system is well established in the literature as a sensitive measure of performance for left-hemisphere, right-hemisphere stroke patients and traumatic brain injured individuals [23,24].

**Table 3.** **Computerized-revised token test multidimensional scoring system.**

**Score**	**Type of response**	**Description of response**
15	Complete	All elements are accurate

14^†^	Rehearsal	Subject repeats command

13	Delay	All elements are accurate but delayed

12	Immediate/impulsive	Response intimated before command completed

11	Self-correct	Self-correct without external prompts

10	Reversal	Elements in two part command are reversed

9	Repeat	Repetition of command

8	Cue	Response of command after unsuccessful repeat

7	Error	Incorrect selection/manipulation

6	Perseveration	Response similar to the previous command

5	Rejection	Rejection of the command

4^†^	Unintelligible/differentiated	Does not attempt task

3^†^	Unintelligible/undifferentiated	Similar to the previous command

2	Omission	Omission of part of the command

1	No response	No response

^†^Responses not scored by the program software.

A score of 15 reflected an accurate and prompt response, and a score of 13 was an accurate but delayed score. The delay reflected the time it would take for the computer program to repeat the test sentence a second time. The mean duration of each spoken sentence was 4.26 s. Therefore, if the participant took longer than 4.26 s to respond to the test sentence, the response would be recorded as delayed. A score of 12 indicated that the participant initiated movement of the mouse to respond before the test sentence was completed reflecting an impulsive response. A correct response on the C-RTT was a score of 15 or 13 score; any score less than 13 was recorded as an error.

Each of eight linguistic elements within a sentence were scored by the software, which generated a mean score for each sentence and an overall mean score for the ten sentences in the subtest. The scoring program also produced an ES for each sentence and an overall ES for the subtest sentences. The ES is a ratio, automatically calculated for accuracy and response time. The scoring system captured the response dimensions of accuracy, responsiveness, completeness, promptness and efficiency, and permitted analysis of an individual’s processing of spoken sentences that a plus/minus scoring system does not [15]. The average words per minute per sentence was 183, and the average syllables per minute was 210. This subtest required less than 10 min to administer and thus did not add a significant amount of time to the testing process.

### Statistical analysis

A preliminary two-factor (between-subjects) ANOVA was conducted to compare the main effects of group membership and sex membership and the interaction effect between sex and group membership on the ES. Group membership might contribute to the ES, but the effect might be different across sexes. No significant main effect of sex was observed and sex did not interact significantly with subject groups (p > 0.05), therefore will not be a significant contributor to additional analyses. Although a significant main effect of subjects groups was observed, a Levene’s test for equality of variances for the between-groups effect (*F* [3, 56] -3.215, p = 0.030) was significant and found to violate test assumptions for the present analysis. Owing to this violation, all assessment of between-groups effect was done using the distribution-free Mann–Whitney U test. To examine the difference in the frequency of types of scores on the C-RTT, a Chi-square Goodness of Fit test was conducted. A stepwise multiple regression analysis was also done to examine how well performance on the ImPACT test composite scores could predict performance on ES of the C-RTT. The predictor variables for a regression analysis were the following: verbal memory, visual memory, visual motor speed, reaction time composite and impulse control.

## Results

All participants in the control group and PC group completed the battery of tests producing valid testing scores. Mann–Whitney t-test showed no differences between the groups for gender, age, years of education and number of hours slept prior to testing. However, C-RTT ES, ImPACT visual memory, verbal memory, visual motor speed, impulse control, reaction time composite scores, total symptom score and history of concussion were statistically different between the two groups.

### Demographic analysis

No statistical effect on C-RTT was identified when controlling for gender. As a result gender was not considered in further analysis. The HC group (n = 30; 22 men and 8 women) ranged in age from 17 to 26 (M = 19.47, SD = 1.80), compared with the PC group (n = 30; 22 men and 8 women) who ranged in age from 18 to 23 (M = 19.80, SD = 1.4). A t-test showed no statistically significant difference in ages between the two groups, p = 0.426. The HC group’s years of education, excluding kindergarten, ranged from 10 to 16 (M = 12.53, SD = 1.79), compared with the PC group who ranged in years of education from 12 to 15 (M = 13.13, SD = 1.01). A t-test showed no statistically significant difference in ages between the two groups; p = 0.063. The hours of sleep the night prior to testing for the HC group ranged from 6.0 to 10.0 (M = 7.44, SD = 1.05), and for the PC group, the range was from 5.0 to 12.0 h (M = 7.30, SD = 1.52). A t-test showed no statistically significant difference in the number of hours sleep the night prior to testing between the two groups, p = 0.749. A Mann–Whitney test indicated that the history of concussion between groups was found to be statistically significant between the two groups. HC mean number of concussions = 0.17, SD = 0.461, PC mean number of concussions = 0.77, SD = 0.971, df = 4.438, p = 0.004.

The PC participants were tested within a range of 1–12 days postinjury (M = 3.83, SD = 2.6). For analysis purposes to reflect potential recovery periods the time of testing PC for the PC group was divided into three day-groups, 0–3 days post (n = 19 subjects), 4–7 days post (n = 9 subjects) and 8–12 days post (n = 2 subjects). An ANOVA analysis showed no statistical difference between the three day-groups for the composite scores on the ImPACT test; verbal memory p = 0.939, visual memory p = 0.246, visual motor speed p = 0.828, reaction time p = 0.863, impulse control p = 0.512. Total symptom score p = 0.937.

### Performance difference between group on Subtest VIII-C-RTT

A Mann–Whitney test was conducted to determine whether the ES differed between PC and HC groups. The analysis indicated that the HC group performed better than the PC group, indicating the HC group made more correct and prompt responses than the PC group. The ES on the Subtest VIII of the C-RTT was statistically greater for the HC group (Mdn = 13.06) than for the PC group (Mdn = 12.68), U = 290.5, p = 0.018.

### Distribution of C-RTT score types between groups

Table 4 presents the occurrence of various response types which differed noticeably between groups.

**Table 4.** **Frequency counts of Computerized-Revised Token Test Subtest VIII scores by type for the healthy control and postconcussion groups.**

**Scores**	**15**	**13**	**12**	**10**	**9**	**7**	**6**	**5**	**Total**
Healthy controls	2164	131	54	4	0	25	7	15	2400

Postconcussion	1789	203	286	2	32	66	4	18	2400

Total number of sentence elements scored were 2400: that is, 8 elements per sentence, 80 sentence elements per participant, times 30 participants, equals 2400 elements.

A Chi-square test of independence was calculated comparing the frequency of occurrence of specific scores for the C-RTT Subtest VIII in the HC group and the PC group. The HC group had significantly higher number of 15 scores (complete, where all elements were accurate), and the Chi-square test indicated that the relationship between these variables was significantly different: *(x^2^)* (1, 60) = 32.389, p < 0.0001. The PC group produced approximately twice the number of 13 scores (delay, all elements are accurate but delayed), and the Chi-square test indicated that the relationship between these variables was also significantly different: (x^2^) (1, 60) 24.352, p < 0.0001. The PC group made approximately five times the number of 12 scores (error, impulsive), and a Chi-square test indicated that these variables were significantly different: (x^2^) (1, 60) = 142.490, p < 0.0001.

### Distribution of C-RTT score types between initial & second sentence phrase

The first grammatical phrase of the sentence included the elements *verb + size + color + shape* (put the small red circle…), and the second phrase included the elements *place + size + color + shape* (…to the left of the large green square). The PC group made fewer correct responses (scores of 15 or 13) during the second grammatical phrase of the sentences (962) than during the first grammatical phrase of the sentences (1030). Similarly the HC group made fewer correct responses during the second grammatical phrase of the sentence (1130) than during the first grammatical phrase of the sentences (1169). Furthermore, the PC group made fewer correct responses on both phrases (1992) compared with the HC group (2299) as presented in Table 5.

**Table 5.** **Frequency counts of correct responses for grammatical phrase 1 and phrase 2 of the Computerized-Revised Token Test Subtest VIII scores for the healthy control and postconcussion groups.**

**Participant groups**	**Grammatical phrase I**	**Grammatical phrase 2**
Healthy control group	1169	1130

Postconcussion group	1034	966

A Chi-square test of independence was calculated comparing the frequency of correct responses (15 or 13) during the first and second phrase, and the analysis showed there was a statistically significant difference: (x^2^) (2, 60) = 1.63, p < 0.05, indicating that more correct responses were made on the first phrase than the second for both groups.

### Relationship between the performance on the C-RTT & ImPACT composite scores

A stepwise multiple regression analysis was computed to determine if any of the five ImPACT composite scores (verbal memory composite, visual memory composite, visual motor speed composite, impulse control score and reaction time composite) predicted ES performance on the C-RTT within each subject group. The results demonstrated that visual reaction time was the only composite score that was entered into the regression equation, having achieved significance at α = 0.05 (R = -0.411, adjusted R^2^ = 0.140; p = 0.012) for the PC group. No ImPACT variables were significantly related to C-RTT ES for the HC group. It should be noted that while the PC group mean for reaction time composite score fell in the low normal range, according to the test norms, one subject’s score was in the borderline range and four subjects’ scores were clearly impaired.

The output from the regression analysis included C-RTT ES scores that were adjusted for the influence of the ImPACT reaction time composite scores. A follow-up one-way ANOVA on the HC versus PC groups was accomplished using ES scores adjusted for ImPACT reaction time composite score for the PC group (note: the HG scores were not adjusted for reaction time composite score because they were not significantly related to the ES scores). Results indicated that there continued to be a significant difference between the groups F(1, 58) = 5.502, p < 0.001). A large effect size was associated with this analysis (partial eta squared = 0.211). Levene’s test for heterogeneity of variance was not statistically significant using the adjusted scores for the PC group.

## Discussion

This study was designed to investigate the auditory comprehension performance of a group of athletes who had experienced a concussion (PC) to a similar group of HC. Prior research by Murdoch and Theodoros suggested a need to examine auditory comprehension within a targeted and homogenous sample of individuals with mTBI such as young athletes [8]. This study is the first that reports a statistically significant difference in auditory comprehension between a recently injured sample of PC athletes and a group of matched healthy individuals.

The only demographic data that indicated a difference between the two groups was history of concussion. Gender, age, years of education and hours of sleep the night before testing were not statistically different between the groups. The non-TBI literature suggests a positive correlation with the number of hours slept prior to testing. For example, a meta-analysis of the influence of reduced number of hours of sleep showed a negative influence on cognitive test performance, executive functioning and behavioral problems in children [25]. A recent study of high school and collegiate athletes showed a significant poorer performance on baseline ImPACT test in participants who reported sleeping less than 7 h the night prior to testing [26]. The participants in both groups in the present study reported an average number of hours slept the night before testing greater than the 7-h threshold. The evolving literature suggests the hours of sleep prior to testing should be considered as a factor in not only interpreting the test results but in treatment planning.

The analyses showed that there was a statistically significant difference in the number of reported concussions suffered between the two groups. The PC group reported more concussions prior to the current concussion than did the participants in the HC group. This finding suggests potential support for the poorer performance of the PC group on the C-RTT was due to their history of concussion. Iverson, Gaetz, Lovell and Collins found that athletes with a history of multiple concussions performed poorer on memory testing than athletes with a single concussion at 2 days PC [27]. Also athletes with multiple concussions reported a significantly higher rate of symptoms PC. While none of the participants in the HC group reported a brain injury within the previous 13 months, as a group they did report a history of previous concussion prior to the threshold of 13 months prior to testing and still performed better than the PC group on the C-RTT. Certainly, the history of concussion requires further investigation.

Also the time of testing PC in the PC group was not a factor. While the test time postonset could potentially be a factor with a larger group of concussed individuals, it was not so with this group of concussed individuals. As a group they continued to show reduced performance on the C-RTT days postonset. Reports in the literature suggest that a large percentage of concussed athletes shows a significant reduction in cognitive test performance within 10–14 days [28]. The present PC group was testing on average well short of the 10–14 day time frame.

As a group, the HC performed statistically better on the auditory comprehension task than the PC group. The PC group made statistically more error responses, were slower in responding and made more impulsive errors than the group of healthy individuals (Table 4). The use of a multidimensional scoring system permitted the documentation of not only prompt and correct responses, but also delayed correct responses and impulsive responses. The use of a multidimensional scoring system permits a fine-grained analysis of the nature of a participant’s response that a plus–minus scoring system does not. It should be noted that the poorer performance of the PC group was not simply the result of slow reaction time, as demonstrated by the analysis that adjusted for visual reaction time. We hypothesize that instead their performance was due to a combination of inefficient auditory-language processing and impulsive responses. The performance for the PC group is consistent with the performance of individuals with mild traumatic brain injury and similar to those with mild aphasia when responding to spoken sentences [9,17,29].

To better understand the breakdown in processing the spoken sentences in the C-RTT an examination of the responses to the individual linguistic elements may offer some useful clinical information. While overall, the PC group made fewer correct responses than the group of healthy individuals, the data in Table 5 show that both groups made fewer correct responses on the second half (*place + size + color + shape*) of the sentence than during the first grammatical phrase of the sentence (*verb + size + color + shape*). The PC and HC participants as groups were more accurate in responding to the linguistic elements in the first phrase of the message (*verb + size + color + shape*) than when responding to elements in the second portion of the message (*place + size + color + shape*). One possible explanation for this similar pattern of performance is that holding the first phrase in WM until the second phrase is processed may exceed the capacity of WM and not permit the integration of both pieces of information into LTM and, therefore, resulting in more error responses. While this experiment was not designed to investigate the theoretical nature of processing auditory information, this analysis provides a starting point for future research. The fact that both the HC and PC participants as a group showed a similar pattern of processing, albeit with different error rates, a comparison with a nonathlete population might shed some light on the potential long-term impact of exposure to concussion present in both groups.

For the linguistically demanding C-RTT task, the fleeting nature of the auditory signal shows that an assessment at a more demanding level of processing provides clinically relevant information that cannot be assumed from the non-auditory ImPACT test alone. The stepwise multiple regression analysis indicates a lack of a significant relationship between the combined ImPACT test composite scores and the ES of the C-RTT Subtest VIII with the exception of reaction time composite score. Reaction time finding suggests relatively little variance in auditory comprehension of sentences can be accounted for by the visually presented tasks and stimuli of the ImPACT. Also this analysis suggests that the C-RTT task is assessing a different functional behavior than the ImPACT test given the lack of relationship to the other composite scores. However, the moderate relationship of reaction time and the ES suggests the importance of assessing reaction time in suspected concussed individuals.

These results provide valuable clinical information for a well-defined group of concussed individuals. First, a sport-related concussion impacts performance across a number of cognitive-communicative parameters. The addition of Subtest VIII of the C-RTT to the clinical assessment battery permits formulation of recommendations about participants’ auditory comprehension based upon the objective assessment of their accuracy, efficiency and impulsivity in following spoken sentences. Furthermore, a dual modality approach to assessment is a more prudent approach to the assessment of PC cognitive-communication behavior than a single visual modality instrument such as the ImPACT test.

The dysfunction of processing spoken sentences shown in these results may potentially impact the individual’s performance in academic, play, social, activities of daily living and work situations. Frequently concussed individuals report experiencing difficulty following classroom lectures and recalling lecture material days and weeks PC. Management of a concussion requires the athlete and their instructors/coaches be made aware of the presence of cognitive-communicative difficulties such as difficulty understanding spoken sentences. While the ImPACT test is a valuable tool for the assessment, treatment and monitoring of an individual’s recovery, additional instruments such as the C-RTT will provide valuable information for clinical decisions made by the practitioner.

In summary, the research question asked if the ES measure on Subtest VIII of the C-RTT reflected a dysfunction in processing sentences presented auditorially for a group of acutely concussed individuals compared with an HC group. As a group, acutely concussed individuals showed statistically significantly greater-difficulty in processing spoken sentences than did healthy individuals. Clinicians should make return to the classroom and to play recommendations based on a multifaceted clinical assessment, including a digitized auditory sentence comprehension test. A digitized format reduces the variability in speech rate frequently present in live voice presentations of auditory test tasks [30]. Furthermore, in cases of identified auditory comprehension processing difficulties, relevant classroom modifications via Section 504 of the Rehabilitation Act of 1973 may be made to facilitate the student athlete’s recovery from a concussion. Future studies using the newly released Pediatric ImPACT test, which uses spoken instructions to the participant, in combination with the C-RTT may reveal the prevalence and nature of auditory processing dysfunction in the pediatric population with sports-related mild traumatic brain injury.

## Conclusion

The statistical analysis indicates that auditory comprehension of the spoken sentences presented is dysfunctional in individuals with an acute sport-related concussion when compared with a matched group of healthy individuals. Individuals with a concussion made fewer correct responses than the healthy individuals. Furthermore, even when they responded correctly, they were delayed in responding and made more impulsive responses compared with healthy individuals. The statistical analysis also indicates that the ImPACT reaction time composite score and the ES from Subtest VIII of the C-RTT are significantly correlated. However, when reaction time was controlled for, the PC group’s performance on the C-RTT remained statistically different for the HC group. The lack of a predictive relationship between the other ImPACT composite scores and C-RTT suggests these two assessment tools are measuring different cognitive-communication processes. The addition of a computerized auditory comprehension test, combined with using a multidimensional scoring system provides a detailed analysis of auditory cognitive-communication processing of spoken sentences. This information will add a significant piece of data to consider in the diagnosis and treatment of concussed individuals.

## Limitations

The history of concussion may play a role in the poorer performance of the PC group. As a group these individuals entered the study with apparent brain dysfunction present prior to their current concussion. To address this possibility a comparison between a matched group of individuals with not reported history of concussion should be investigated.

## Future perspective

The evolving investigation of the diagnosis, treatment, management and prevention of sports-related concussion offers a variety of challenges the least of which is the sports culture. Future scientific findings may or may not benefit the athlete if the culture of ‘playing hurt’, for example, does not change. Given the constraints of this culture the recent findings from the fields of brain imaging and metabolic analysis offer an exciting phase in this evolving area of research. The behavioral assessment tests for PC individuals similar to those reported in this current paper will become more precise and efficient in identifying behavioral markers sensitive enough to identify and monitor individuals over time. This goal must be accomplished given the economic challenge of providing each concussed individual an assessment via diffuse tensor imaging or magnetic resonance spectroscopy. Studies will, for example, investigate the nature of recovery of auditory comprehension behavior from a concussion over time. These behavioral assessment tests may be highly correlated with both imaging biomarkers and metabolic biomarkers. Assessment of auditory comprehension behavior should play a more prominent role in the development of return-to-classroom and return-to-play decision-making.

**Summary points**

- Auditory comprehension plays a significant role in social, educational and employment functions and, therefore, demands significant attention to the assessment and treatment of concussed individuals.
- No data exist that assess the impact of a sports-related concussion on auditory comprehension or if the impact on auditory comprehension is different from auditory comprehension in healthy control participants.
- The current study shows that individuals with acute concussion perform poorer than healthy control participants in responding to spoken sentences.
- The present findings suggest that the impact of a concussion is not reflected equally across the visual and auditory modalities given the weak relationship between performance on visually presented cognitive tasks and auditory presented tasks.
- The present findings suggest that the pattern of errors produced in the auditory task by concussed and nonconcussed individuals is similar but depressed in the concussed individuals.

## Appendix

Test administration instructions for the Computerized-Revised Token Test (C-RTT) Subtest VIII.

Participants completed the C-RTT-Subtest VIII individually with a trained test administrator present. Each participant received the same test instructions when administering Subtest VIII. The test instructions included a brief description of Subtest VIII first, followed by a short assessment to test whether participants are color-blind using C-RTT test items. Participants were asked to point to any object that is green, red and blue. Last, the test administrator provided clear verbal directions for completing the short test. The instructions included the following information: ‘This task will assess your ability to understand ten simple two-part commands. The test takes about 5 min to complete. As soon as I start the test, the computer will begin to give you instructions. This program is highly sensitive, so please do not touch the mouse until each command is completed. Once the spoken command is completed, you are to follow the command by clicking and dragging the desired token next to the other desired token. Once you complete the command then return the mouse to the target at the bottom of the screen. Once the mouse is returned to the target, the next command will be given to you. The tokens differ by size, shape and color. Are you ready?’
